# 1284. Suppressed Switch to DTG/3TC 2-Drug Regimen Vs. BIC- or DTG-Based 3-Drug Regimens

**DOI:** 10.1093/ofid/ofac492.1115

**Published:** 2022-12-15

**Authors:** Gerald Pierone, Jennifer S Fusco, Laurence Brunet, Vani Vannappagari, Supriya Sarkar, Cassidy Henegar, Jean A van Wyk, Andrew Zolopa, Gregory P Fusco

**Affiliations:** Whole Family Health Center, Vero Beach, Florida; Epividian, Inc., Durham, North Carolina; Epividian, Inc., Durham, North Carolina; ViiV Healthcare, Research Triangle Park, North Carolina; ViiV Healthcare, Research Triangle Park, North Carolina; ViiV Healthcare, Research Triangle Park, North Carolina; ViiV Healthcare Limited, Brentford, England, United Kingdom; ViiV Healthcare, Research Triangle Park, North Carolina; Epividian, Inc., Durham, North Carolina

## Abstract

**Background:**

Real-world effectiveness of fixed dose dolutegravir/lamivudine (DTG/3TC) two-drug regimens (2DR) during the first 24 months of availability in the US was compared to common three-drug regimens (3DRs) among suppressed antiretroviral therapy (ART)-experienced people living with HIV (PLWH).

**Methods:**

Suppressed (viral load [VL] < 200 copies/mL) PLWH initiating DTG/3TC 2DR, bictegravir (BIC)-3DR, or DTG-3DR between 01MAY2019 and 31OCT2020 in the OPERA^®^ Cohort were followed until 30APR2021 (potential for ≥6 months of follow-up). Univariate Poisson regression (incidence rates) and Cox proportional hazards marginal structural models were employed to assess confirmed virologic failure (2 viral loads [VLs] ≥200 copies/mL) or regimen discontinuation.

**Results:**

Overall, 8037 PLWH were included in the analysis (Table). Virologic failure incidence rates were low, ranging from 0.66 (DTG/3TC) to 1.78 (DTG 3DR) per 100 person-years. Compared to DTG/3TC, only DTG 3DR was associated with an increase in the hazard of virologic failure. Discontinuation incidence rates ranged from 8.30 (BIC 3DR) to 24.9 (DTG 3DR) per 100 person-years. The discontinuation hazard was 69% greater with DTG 3DRs and 49% lower with BIC 3DRs compared to DTG/3TC. Regardless of regimen, most discontinuers were suppressed (VL< 200 copies/mL) at the time of discontinuation (DTG/3TC 2DR: 96%, BIC 3DR: 94%, DTG 3DR: 93%; all p >0.05). Discontinuations following an adverse diagnosis/side effect were uncommon with DTG/3TC 2DR (3%) and DTG 3DR (4%, p=0.5), and higher with BIC 3DR discontinuation (7%, p=0.02). The most common reason for DTG 3DR discontinuations was regimen simplification (21%); no reason was given for >50% of the discontinuations in each group.

**Figure:**
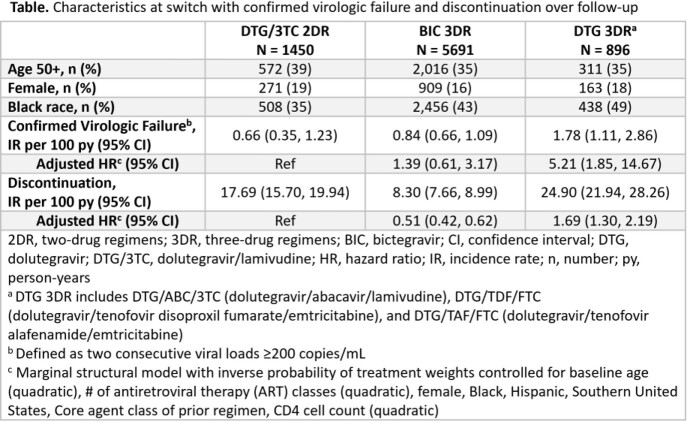

**Conclusion:**

Among ART-experienced, virologically suppressed PLWH, virologic failure was rare after switching to DTG/3TC 2DR, BIC 3DR or DTG 3DR. Most discontinuations were not attributed to the treatment (i.e., loss of suppression, adverse diagnosis, side effects), suggesting other reasons for discontinuation despite high levels of suppression and tolerability.

**Disclosures:**

**Gerald Pierone, Jr., MD**, Gilead: Grant/Research Support|GSK-VIIV: Grant/Research Support **Jennifer S. Fusco, BS**, AIDS Healthcare Foundation: Client of my employer|EMD Serono: Client of my employer|Gilead Sciences: Client of my employer|Janssen: Client of my employer|Merck & Co.: Client of my employer|TheraTechnologies: Client of my employer|ViiV Healthcare: Client of my employer **Laurence Brunet, PhD**, AIDS Healthcare Foundation: Client of my employer|EMD Serono: Client of my employer|Gilead Sciences: Client of my employer|Janssen: Client of my employer|Merck & Co: Client of my employer|TheraTechnologies: Client of my employer|ViiV Healthcare: Client of my employer **Vani Vannappagari, MBBS, MPH, PhD**, ViiV Healthcare: I am full time employee of ViiV Healthcare and receive GlaxoSmithKline stock as part of my compensation package|ViiV Healthcare: Stocks/Bonds **Supriya Sarkar, PhD, MPH**, ViiV Healthcare: Salary|ViiV Healthcare: Stocks/Bonds **Cassidy Henegar, PhD, MSPH**, GlaxoSmithKline: Stocks/Bonds|ViiV Healthcare: full-time employee **Andrew Zolopa, MD**, ViiV Healthcare: full time employee|ViiV Healthcare: Stocks/Bonds **Gregory P. Fusco, MD, MPH**, AIDS Healthcare Foundation: Client of employer|EMD: Grant/Research Support|Gilead Sciences: Client of employer|Janssen: Client of employer|Merck & Co.: Client of employer|Theratechnologies: Client of employer|ViiV Healthcare: Client of employer.

